# A Web-Based Serious Game for Health to Reduce Perioperative Anxiety and Pain in Children (CliniPup): Pilot Randomized Controlled Trial

**DOI:** 10.2196/12431

**Published:** 2019-06-01

**Authors:** Connor Buffel, June van Aalst, Anne-Marie Bangels, Jaan Toelen, Karel Allegaert, Sarah Verschueren, Geert Vander Stichele

**Affiliations:** 1 MindLab Interactive AI Inc Edmonton, AB Canada; 2 Division of Nuclear Medicine and Molecular Imaging Department of Imaging and Pathology KU Leuven Leuven Belgium; 3 Department of Development and Regeneration KU Leuven Leuven Belgium; 4 Division of Neonatology Department of Pediatrics Erasmus Medical Center–Sophia Children’s Hospital Rotterdam Netherlands; 5 MindBytes BVBA Merksplas Belgium

**Keywords:** serious games for health, behavior change, perioperative pain, perioperative anxiety, pediatric, ambulatory surgery

## Abstract

**Background:**

As pediatric ambulatory surgeries are rising and existing methods to reduce perioperative anxiety and pain are lacking in this population, a serious game for health (SGH), CliniPup, was developed to address this unmet need. CliniPup was generated using the SERES framework for serious game development.

**Objective:**

The goal of the research was to clinically evaluate CliniPup as an adjunct therapy to existing pharmacological interventions aimed at reducing perioperative anxiety and pain in children undergoing ambulatory surgery.

**Methods:**

CliniPup was evaluated in a prospective randomized controlled pilot trial in 20 children aged 6 to 10 years who underwent elective surgery and their parents. Study participants were randomly assigned to the test (n=12) or control group (n=8). Children in the test group played CliniPup 2 days prior to surgery, and children in the control group received standard of care. On the day of surgery, pediatric anxiety was measured with the modified Yale Preoperative Anxiety Scale and parental anxiety was assessed with the State-Trait Anxiety Inventory. Pediatric postoperative pain was assessed by the Wong-Baker Faces Pain Rating Scale. Child and parent user experience and satisfaction were also evaluated in the test group using structured questionnaires.

**Results:**

Despite the small sample, preoperative anxiety scores were significantly lower (*P*=.01) in children who played CliniPup prior to surgery compared to controls. Parental preoperative anxiety scores were also lower in the test group (*P*=.10) but did not reach significance. No significant differences were observed in postoperative pain scores between groups (*P*=.54). The evaluation of user experience and satisfaction revealed that both children and parents were satisfied with CliniPup and would recommend the game to peers.

**Conclusions:**

Results of the pilot trial introduce CliniPup as a potentially effective and attractive adjunct therapy to reduce preoperative anxiety in children undergoing ambulatory surgery with a trend toward positive impact on parental preoperative anxiety. These results support the use of the SERES framework to generate an evidence-based SGH that results in positive health outcomes for patients. Based on these preliminary findings, we propose a research agenda to further develop and investigate this tool.

**Trial Registration:**

ClinicalTrials.gov NCT03874442; https://clinicaltrials.gov/ct2/show/NCT03874442 (Archived by WebCite at http://www.webcitation.org/78KZab8qc)

## Introduction

### Background

Ambulatory surgeries are increasing at a significant rate, and procedures are associated with high levels of perioperative pain and anxiety [1-4]. This is particularly true in children, with 40% to 60% experiencing high levels of anxiety on the day of surgery, and more than 30% experiencing moderate to severe postoperative pain [1,2,4,5]. This leads to both acute and long-term physical and psychosocial outcomes [5-7].

The current management of perioperative anxiety and pain is particularly difficult in children and is considered inadequate due to the limitations of existing interventions [6,8,9]. Specifically, the use of alternative nonpharmacological interventions to reduce anxiety is limited by cost, time restrictions, availability, and efficacy [6,7,9,10]. In addition, pharmacological interventions are limited by adverse effects, inconsistent prescribing practices, and poor parental assessment and understanding of pain [11-18]. Therefore there is a need to develop additional interventions to better address perioperative anxiety and pain.

Digital interventions have the potential to prepare, educate, and/or distract children, which may result in health outcome benefits such as reductions in perioperative anxiety [7,19-22]. MindBytes is focused on empowering and educating individuals using interactive digital tools and developed a serious game for health (SGH), CliniPup, to prepare children and their parents for surgery [23].

CliniPup has the potential to address limitations of existing nonpharmacological interventions as it is relatively low cost, requires minimal time and resources, and could be widely deployed. Moreover, it has the potential for synergy as an adjunct to pharmacological interventions by preparing parents for postoperative pain management. In addition, it has the potential to address gaps of distraction techniques by preparing both children and parents in a fun and intuitive manner [23]. Although it is clear that preparing both children and parents is vital, this research focused mainly on children due to the challenge of addressing child and parental learning objectives in a single SGH [6,7,13,24].

This study evaluated CliniPup’s usability, safety, and effectiveness in a pilot trial with the target population.

### Objective

The objective of this research was to evaluate CliniPup’s usability and clinical impact in comparison to the standard of care in a pilot trial.

## Methods

### Pilot Trial

CliniPup’s impact on perioperative pain and anxiety in children was evaluated in a pilot clinical trial with 20 children aged 6 to 10 years who were with their parents and undergoing ambulatory surgery. Several secondary outcomes were also evaluated in the trial such as parental anxiety and SGH usability.

### Trial Design

**Figure 1:**
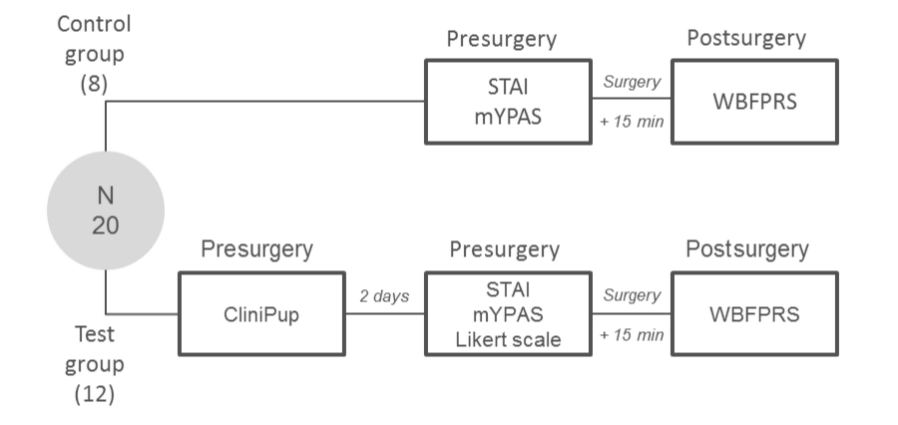
Study design flowchart. STAI: State-Trait Anxiety Inventory; mYPAS: modified Yale Preoperative Anxiety Scale; WBFPRS: Wong-Baker Faces Pain Rating Scale.

The study was a prospective, randomized, 2-armed controlled pilot study conducted at 2 institutions in Belgium (RZ Tienen, Campus Mariendal and RZ Tienen, Medisch Centrum Aarschot) from April to May 2016. Study participants were assigned to a test group (n=12) or a control group (n=8) using a block randomization technique dependent on the week of contact by the researcher (eg, one week participants were randomized to test group and the following week, participants were randomized to the control group). Children in the test group played CliniPup 2 days prior to surgery. On the day of surgery, children’s anxiety was measured with the modified Yale Preoperative Anxiety Scale (mYPAS) and parental anxiety was assessed with the State-Trait Anxiety Inventory (STAI). Pediatric postoperative pain was assessed by means of the Wong-Baker Faces Pain Rating Scale (WBFPRS). The testing protocol for the control subjects only differed in that they did not receive an intervention, whereas the assessments remained the same (except for the Likert scale; Figure 1).

### Participants

A total of 32 children were contacted for inclusion in the study from March to April 2016 from 2 centers in Belgium. Every other week the subjects were included in either the test group or the control group. Eligible participants and their parents for the test group were recruited 2 days before surgery through patient lists of scheduled operations in the surgical day center of RZ Tienen or Medisch Centrum Aarschot in consultation with the doctors. Two days before surgery, parents were contacted by telephone and invited to participate with their child in the study. If they were interested in participation, an email was sent with a link to the CliniPup game. Parents were asked to play the game together with their child. Study information for the parent and child and consent and assent papers were sent via email.

Participants in the control group were recruited at the hospital prior to surgery. Inclusion criteria were (1) aged 6 to 10 years, (2) parents have signed an informed consent, (3) children have given assent, and (4) children and parents understand and speak the Dutch language. Exclusion criteria were (1) children who have been diagnosed with a mental illness, (2) children who have been diagnosed with a developmental delay, (3) children who have a history of an affective disorder, and (4) children with an American Society of Anesthesiology physical status greater than II.

Written informed consent was obtained from 20 parents and assent from 20 children included in the study. The Medical Ethics Committees of UZ Leuven, KU Leuven, and RZ Tienen in Belgium approved all study procedures in advance (study number S58541).

### Interventions

The test group accessed CliniPup online and played at home 2 days prior to surgery, and the control group received no intervention (ie, received standard of care).

### Outcomes

#### Primary Outcomes

##### Preoperative Anxiety

After registration and admission at the hospital, study information was explained to the parent and the child. After signing the consent and assent papers, the preoperative anxiety of the child was measured by the researcher using the mYPAS. The mYPAS is a validated structural observational scale consisting of 27 items in 5 domains (activity, vocalizations, emotional expressivity, state of arousal, and use of parent) of behavior indicating anxiety in young children [25]. After the assessment, a total adjusted score ([activity score/4 + vocalizations score/4 + emotional expressivity score/4 + state of arousal/4 + use of parent score/4] * 100/5) was calculated [25].

Aligned with the institution guidelines, some of the children were given premedication. However, assessment of preoperative anxiety was always completed before the administration of the premedication.

##### Postoperative Pain

Children were asked to scale their pain using the WBFPRS once they were awake and responsive (15 minutes after they were back in their room). The WBFPRS is used to assess pain in children and help them communicate about it [26]. The WBFPRS, which is an auto-evaluation scale, has six faces representing no pain (0) to worst pain ever (5) [27].

#### Secondary Outcomes

##### Parental Preoperative Anxiety

After signing the consent and assent papers, parental anxiety was assessed with the STAI, a widely used self-report anxiety-assessment instrument [28]. There are 2 subscales: State Anxiety Scale and Trait Anxiety Scale. The State Anxiety Scale evaluates the current state of anxiety using items that measure feelings of apprehension, nervousness, tension, worry, and so on. The Trait Anxiety Scale evaluates relatively stable aspects of anxiety including general states confidence, calmness, and security. A total STAI score can be calculated by adding all scores. For the anxiety-absent items, the scores should be reversed (19 items of the total 40) [29].

##### User Experience and Satisfaction

In the case of the test group, user experience and satisfaction was also assessed through a questionnaire where parents and children completed a Likert scale for each question. Additionally, parents were asked to what extent they would recommend CliniPup to peers, and a net promoter score (NPS) was calculated [30].

### Statistical Analysis

A descriptive analysis was performed on the results. No formal power calculation was used. In addition, the children were divided in this study into 2 groups, a test group and a control group. A Fisher exact test was used to assess if gender, previous surgery, and the use of premedication were significantly different between the two groups. Additionally, a Wilcoxon rank-sum test (a nonparametric analogue of the *t* test for two independent samples) was performed to test if the distribution of age was the same across both groups. An independent *t* test was used to determine if there were significant differences in parental anxiety scores between the two groups. A Mann-Whitney U test was used to determine if the preoperative anxiety scores and the postoperative pain scores differed significantly between the control and test group. All statistical analyses were performed using SPSS version 23.0 statistical software (IBM Corp) and were 2-sided with a level of significance of .05.

## Results

### Patient Flow

**Figure 2:**
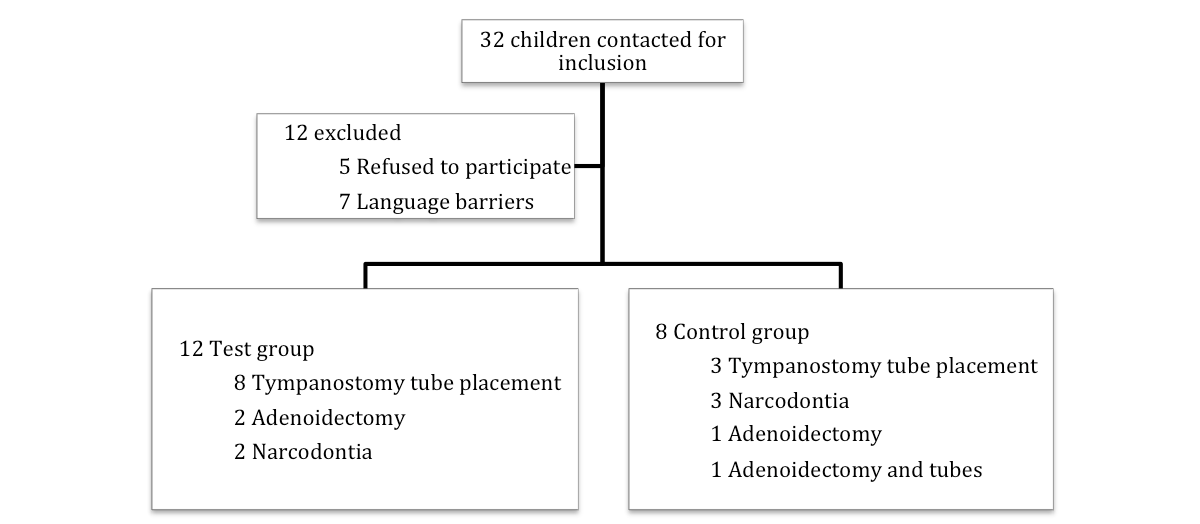
Patient flow diagram.

Every child aged between 6 and 10 years who met the inclusion criteria was invited to enter the study. In total, 32 children were contacted. After exclusions due to language barriers and no interest in participation, 20 children were recruited. Twelve children were included in the test group and 8 in the control group. In the test group, 8 children were scheduled for tympanostomy tube placement, 2 for adenoidectomy, and 2 for narcodontia (dental care that takes place under general anesthesia). In the control group, 3 children were scheduled for tympanostomy tube placement, 3 for narcodontia, 1 for adenoidectomy, and 1 for adenoidectomy in combination with tympanostomy tube placement (Figure 2).

### Baseline Characteristics

Baseline characteristics of study participants are shown in Table 1. There were no significant differences in age, gender, premedication use, and previous surgery experience between the two groups. The distribution of age and gender was the same across the two groups.

### Primary Outcomes

#### Preoperative Anxiety

**Figure 3:**
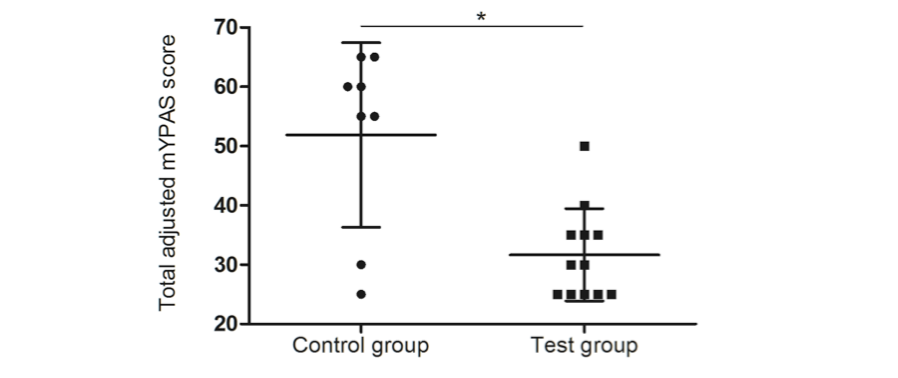
Child preoperative anxiety. mYPAS: modified Yale Preoperative Anxiety Scale.

The researchers measured preoperative anxiety in study participants with the mYPAS observational scale. The mean total adjusted mYPAS score showed a significant difference between the control group and test group (51.88 [SD 15.57] vs 31.67 [SD 7.79], respectively; *P*=.01; Figure 3). This demonstrated that children who played CliniPup before surgery were less anxious than children who did not play CliniPup. No significant correlation was found between the number of times children played CliniPup (1.83 [SD 1.03]) and the total adjusted mYPAS score.

**Table 1:** Baseline study participant characteristics.

Child characteristic		Control group (n=8)	Test group (n=12)	*P* value
Age in years, mean (SD)		6.87 (0.93)	7.49 (1.82)	.52
Gender, male, n (%)		3 (38)	6 (50)	.67
Previous surgery, yes, n (%)		2 (25)	9 (75)	.07
Premedication with midazolam, yes, n (%)		5 (63)	2 (17)	.06
Number of times CliniPup was played, mean (SD)		—^a^	1.83 (1.03)	—
**Surgery type, n (%)**				
	Tympanostomy tube placement	3 (38)	8 (67)	—
	Adenoidectomy	1 (13)	2 (17)	—
	Narcodontia	3 (38)	2 (17)	—
	Adenoidectomy and tubes	1 (13)	0 (0)	—

^a^Not applicable.

#### Postoperative Pain

**Figure 4:**
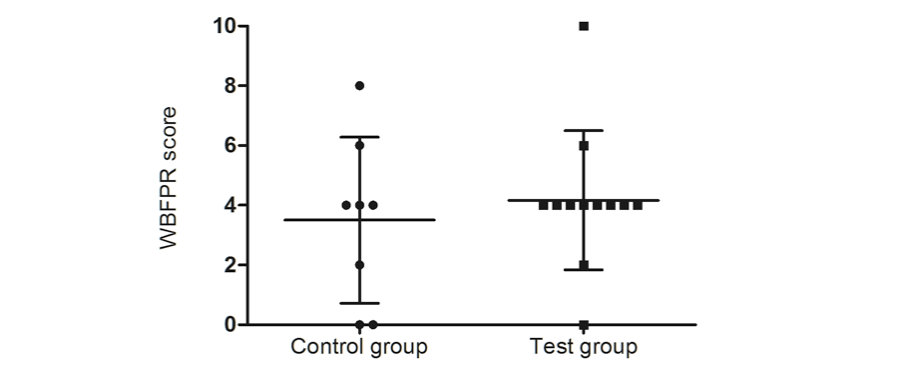
Child postoperative pain. WBFPR: Wong-Baker Faces Pain Rating Scale.

Postoperative pain in study participants was measured with the WBFPRS scale 15 minutes postoperatively. No significant differences were shown in the analysis of the postoperative pain scores between the control and test group (3.50 [SD 2.77] vs 4.18 [SD 2.33], respectively; *P*=.54; Figure 4).

### Secondary Outcomes

#### Parental Preoperative Anxiety

**Figure 5:**
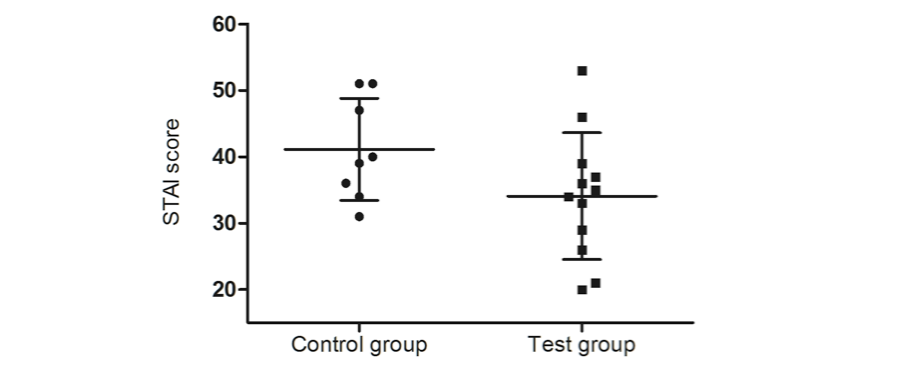
Parental preoperative anxiety. STAI: State-Trait Anxiety Inventory.

Parental preoperative anxiety was measured with the STAI completed by the parent before surgery. No significant difference was found between the control group and test group (41.13 [SD 7.70] vs 34.09 [SD 10.00], respectively; *P*=.10; Figure 5).

#### User Experience and Satisfaction

##### Children

To evaluate CliniPup, we asked children to complete a Likert scale questionnaire. Sixty-seven percent (8/12) of children liked CliniPup very much, 17% (2/12) of children liked CliniPup at least somewhat, and 17% (2/12) were undecided. Children were also asked if they learned anything by playing CliniPup. Forty-two percent (5/12) learned very much, 33% (4/12) learned somewhat, 20% (2/12) were undecided, and 8% (1/12) learned nothing at all. In summary, 75% (9/12) of children learned somewhat or very much from playing CliniPup.

##### Parents

One parent of each child (the parent who played CliniPup together with their child) completed the Likert scale questionnaire and provided information on the extent with which they would recommend CliniPup to peers.

Eighty-three percent (9/12) of the parents strongly agreed that “CliniPup was clear; my child understood everything.” Fifty-eight percent (7/12) of parents strongly agreed and 42% (5/12) agreed that “CliniPup helped to prepare my child for surgery.” Forty-two percent (5/12) of parents strongly agreed and 50% (6/12) agreed that “I received useful information by playing CliniPup together.” Additionally, 42% (5/12) disagreed, and 25% (3/12) strongly disagreed that “I had to give additional information to my child.” Lastly, 17% (2/12) strongly agreed, 33% (4/12) agreed, and 50% (6/12) did not disagree nor agree that they would like something similar to prepare themselves for the surgery of their children.

When asked on a scale from 0 to 10 (very unlikely to very likely) to what extent they would recommend CliniPup to their friends/family/colleagues, 42% (5/12) of parents gave a 10, 25% (3/12) gave a 9, 17% (2/12) gave an 8, and 17% (2/12) gave a 7. These results correspond to an NPS of 67%.

## Discussion

### Principal Findings

The findings of this pilot study suggest that the use of CliniPup, an SGH, may be an effective adjuvant intervention to contemporary management of perioperative anxiety and pain in children undergoing ambulatory surgery and their parents. The results of the pilot trial showed that the test group experienced significantly less preoperative anxiety, as measured by the mYPAS scale, than the control group (Figure 3). In contrast, there were no between-group differences in postoperative pain, as measured by WBFPRS, immediately (15 minutes) after surgery (Figure 4). This was not surprising given that operations (tympanostomy tube placement, adenoidectomy, narcodontia, and adenoidectomy in combination with tube placement) were unequally distributed between the groups. This makes it difficult for a fair and representative comparison of postoperative pain scores between groups.

Considering the secondary outcomes, parents of children in the test group experienced equivalent, with a trend toward reduced, preoperative anxiety compared to parents of children in the control group (Figure 5). In addition to the clinical outcomes, the study determined that the majority of children enjoyed playing CliniPup very much and learned a lot by playing CliniPup. Parents agreed with this sentiment as the majority agreed that CliniPup helped their child prepare for surgery. Moreover, CliniPup’s NPS was considered excellent, demonstrating that parents were likely to recommend this nonpharmacological intervention to others.

By alleviating anxiety in the home setting prior to surgery, this SGH may address challenges associated with current nonpharmacological interventions such as cost, time requirements, and accessibility and availability [7,9,10]. This may, in turn, reduce the long-term psychosocial burden experienced by children with perioperative anxiety and pain [2,24,31,32]. CliniPup may also offer the potential to improve economic outcomes by reducing medical resource use and decreasing hospital discharge times [2,24,31-33]. However, this would require more targeted research to confirm.

CliniPup shows the potential to address the unmet need of children in the perioperative setting by offering an effective, inexpensive, accessible, and evidence-based nonpharmacological intervention to reduce perioperative anxiety and pain in children.

The data collected within this pilot trial support the conclusion that an SGH, when developed using a scientific methodology such as the SERES Framework, may be particularly valuable as an intervention to educate and change behavior in health care settings [34]. This may be particularly true for a pediatric population, but the generalizability of the approach implemented also suggests that this method could be applicable in adult populations as well. In addition, it is conceivable that similar interventions could be developed to realize behavior change and impact health outcomes in other fields such as mental health, rare disease, cardiovascular health, oncology, etc.

The results collected in this pilot trial serve as important inputs to be used in subsequent iterations of the SGH. This was, in particular, one of the key objectives of the pilot trial, in that the findings will be used to inform the next steps of trial design and game development.

### Limitations

The results of this study, however, should be considered as preliminary due to the small sample size and methodological limitations (block randomization, nonblind, inactive control group, two study centers, child and surgical characteristics not controlled for, etc) of a pilot trial. Although the findings of this pilot study are promising, further research is required to validate CliniPup’s efficacy in a pivotal trial that is adequately powered to detect between group differences. Additionally, it may be prudent to include an active control and further control for child and surgical characteristics to limit potential methodological biases. Moreover, it may be valuable to investigate the mechanistic bases for the observed changes in perioperative anxiety, which may differ from hypothesized theories and could inform clinical practice. Finally, methodological changes or adaptations to the SGH CliniPup should be considered to enhance the potential for addressing postoperative pain in addition to preoperative anxiety. For example, it is logical to theorize that the addition of a supplementary digital tool targeted at parents of children undergoing ambulatory surgery could improve outcomes, as this would explicitly address parental fears, a key determinant of child pain and anxiety [23]. Such a tool has subsequently been developed, and methodological modifications will be integrated into an upcoming pivotal study to also evaluate (behavioral) changes in postoperative pain management at home.

### Conclusion

In summary, the results of the pilot study introduce CliniPup as a potentially effective and attractive adjunct therapy to existing interventions aimed at reducing preoperative anxiety in children undergoing ambulatory surgery. The findings also support the validity of the SERES framework for SGH development with respect to achieving positive health outcomes. The results provide important data to inform subsequent development of the SGH and its future clinical validation. Moreover, based on theories applied in the development of the SGH, supplemental components can be integrated to enhance CliniPup’s impact on pediatric perioperative anxiety and pain outcomes. Aligned with this, CliniPup’s safety and efficacy should be evaluated further in a pivotal trial of the optimized SGH.

## Appendix

Multimedia Appendix 1 CONSORT‐EHEALTH checklist (V 1.6.1).

## Abbreviations

mYPAS: modified Yale Preoperative Anxiety Scale
NPS: net promoter score
SGH: serious game for health
STAI: State-Trait Anxiety Inventory
WBFPRS: Wong-Baker Faces Pain Rating Scale
